# Renal Phosphate Wasting Due to Tumor-Induced (Oncogenic) Osteomalacia

**DOI:** 10.7759/cureus.15507

**Published:** 2021-06-07

**Authors:** Eluwana A Amaratunga, Emily B Ernst, James Kamau, Ragarupa Kotala, Richard Snyder

**Affiliations:** 1 Internal Medicine, St. Luke’s University Health Network, Easton, USA

**Keywords:** tumor-induced osteomalacia, mesenchymal tumors, fgf 23, fibroblast growth factor 23, renal phosphate wasting, phosphatonins, mixed connective tissue tumors, hyperphosphaturia, hypophosphatemia

## Abstract

Osteomalacia is a widely prevalent bone disorder that is caused by an imbalance in body calcium and phosphate. Tumor-induced osteomalacia (TIO) is a rare form of osteomalacia that is associated with mesenchymal tumors. It is caused by overproduction of fibroblast growth factor 23 (FGF-23), a hormone involved in phosphate regulation.

A 59-year-old male with a history of factor V Leiden mutation, pulmonary embolism, and deep vein thrombosis was diagnosed with oncogenic osteomalacia in 2008 following laboratory findings significant for low phosphorus and elevated FGF-23 levels. He underwent a resection of a right suprascapular notch mass with the biopsy confirming a phosphaturic mesenchymal tumor. He was maintained on oral phosphorus and calcitriol replacements with a regular follow-up with oncology and nephrology. Eight years later, the patient’s phosphorus levels started declining despite replacement. A repeat test showed FGF-23 levels once again elevated. A whole-body magnetic resonance imaging (MRI) scan showed no significant findings. The patient was continued on oral replacement therapy with a close follow-up. Two years later, urine phosphorus excretion was elevated at 2494 mg per 24 hours with low plasma phosphorus (1.2 mg/dL) and an elevated FGF-23 level of 1005 relative units (RU)/mL. A repeat MRI of the right shoulder revealed a mass in the supraspinatus muscle and another in the spinal glenoid notch. The masses were resected and the biopsy was consistent with a recurrence of the phosphaturic mesenchymal tumor. Follow-up serum phosphate levels remained in the normal range.

FGF-23 plays a critical role in bone mineralization through the regulation of phosphate levels. Overproduction, as seen in mesenchymal tumors, results in hyperphosphaturia, hypophosphatemia, and low calcitriol levels. While the definitive treatment of TIO involves the resection of the mesenchymal tumor, localization of the tumor is often challenging given its small size and slow growth. This leads to delayed diagnosis and treatment. For individuals whose tumor cannot be resected or detected, burosumab is the preferred form of therapy. Interestingly, FGF-23 is shown to have a potential cardiovascular (CV) morbidity and mortality through various mechanisms like activation of myocardial FGF-23 receptors, endothelial dysfunction, inflammation, and altered phosphorus and vitamin D metabolisms. While studies have shown possible FGF-23 effects on CV outcomes in patients with chronic kidney disease, this has not been proven in cases of TIO.

## Introduction

Osteomalacia, characterized by impaired bone matrix mineralization, is a common bone disorder prevalent worldwide. It is caused by an imbalance in body calcium and phosphate, which are primarily regulated by vitamin D [1,2]. While there are numerous etiologies of osteomalacia, tumor-induced osteomalacia (TIO) associated with mesenchymal tumors is a rare occurrence [2-4]. This is due to the overproduction of fibroblast growth factor 23 (FGF-23), a hormone that plays an important role in phosphate regulation [3,5]. Other phosphatonins such as matrix extracellular phosphoglycoprotein (MEPE) and frizzled-related protein 4 (FRP4) may be potential contributors; however, their exact role in TIO remains unclear [3,4].

While the prevalence of TIO is currently unknown, since its first description in 1947 by McCance [6], approximately 500 cases have been reported in the literature [7-9]. The diagnosis is often challenging due to the rarity of this condition as well as its nonspecific presentations, such as bone pain, muscle weakness, and fractures [7,8,10]. Although TIO is typically associated with benign tumors, malignant variants have been described [10-12]. This condition can cause significant impairment in an individual’s quality of life, if not diagnosed in a timely manner, hence the need for increased awareness among health care providers [7].

We report a rare occurrence of TIO in a patient with recurrent mesenchymal tumors. We further highlight the important diagnostic and therapeutic considerations in patients with suspected TIO.

## Case presentation

The patient was a 59-year-old male with a past medical history significant for factor V Leiden mutation, pulmonary embolism, and deep vein thrombosis on warfarin. He was diagnosed with oncogenic osteomalacia in 2008 after presenting with a low phosphorus and an elevated FGF-23 level. A full-body magnetic resonance imaging (MRI) followed by a dedicated MRI of the right shoulder reported a mass in the right suprascapular notch; the patient underwent surgical resection with the biopsy confirming a phosphaturic mesenchymal tumor. After this, he was followed up regularly with oncology and nephrology and was maintained on oral phosphorus replacement as well as calcitriol.

**Figure 1:**
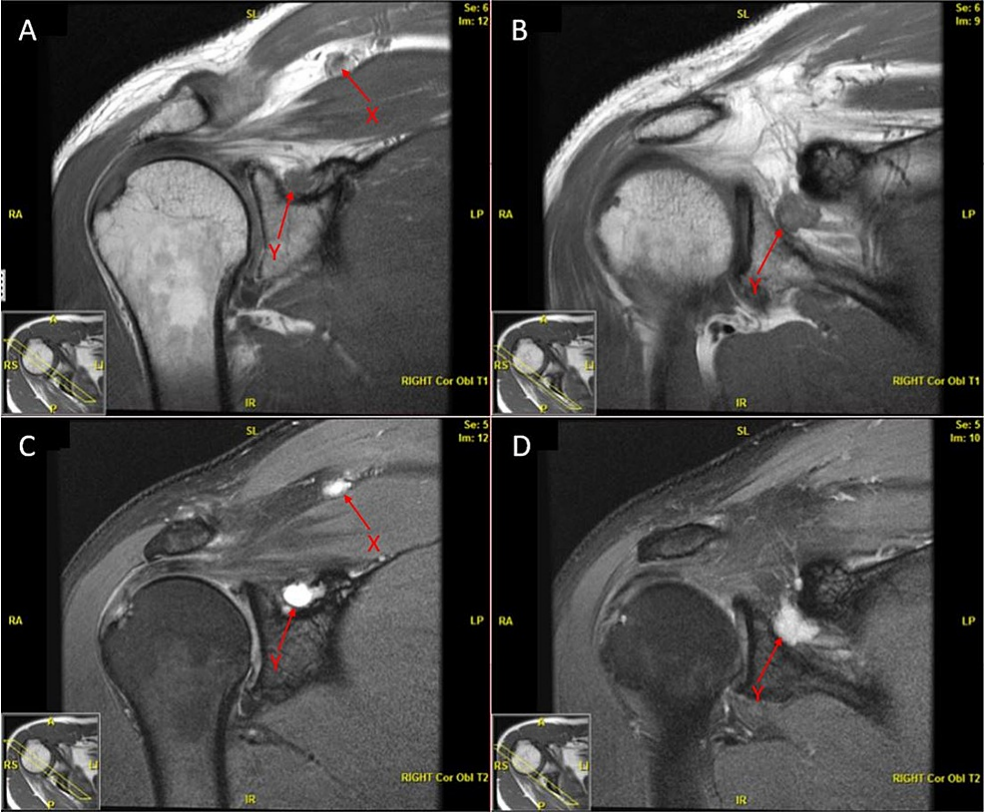
T1-weighted (A, B) and T2-weighted (C, D) MRI images of the right shoulder demonstrating mesenchymal tumors along the superior aspect of the supraspinatus muscle belly (arrow X) and within the suprascapular notch (arrow Y)

Later in 2016, his regular laboratory workup began to show declining phosphorus levels once again with an elevated FGF-23 level of 410 relative units (RU)/mL. At that time, MRI of the soft tissue neck and MRI of the right shoulder did not show any abnormalities. The dual-energy X-ray absorptiometry scan was also normal. The phosphorus levels continued to decrease; a whole-body MRI was ordered that was unremarkable. He was monitored closely until 2018 when he returned to the Mayo Clinic, MN, for further evaluation. Phosphorus excretion in urine was elevated at 2494 mg/24 hours, plasma phosphorus was low at 1.2 mg/dL, and FGF-23 elevated at 1005 RU/mL. 25-hydroxyvitamin D was 35 ng/mL and 1,25-dihydroxyvitamin D was 21 pg/mL. Serum electrophoresis did not reveal any monoclonal proteins. MRI of the right shoulder was repeated and this time it showed two masses: the first located along the superior aspect of the supraspinatus muscle belly and the second located within the suprascapular notch extending into the spinoglenoid notch (Figures 1A-1D). These findings were compatible with recurrent mesenchymal tumors, for which he again underwent surgical resection. A biopsy of the tissue obtained from the right trapezius muscle showed a phosphaturic mesenchymal tumor forming a soft tissue mass involving skeletal muscle. Chromosomal analysis indicated recurrence of the patient’s neoplastic clone with 8 out of 20 metaphases being structurally abnormal 2q, 4q, and 20q.

The patient is followed up closely, in every three to four months, with regular lab monitoring of serum phosphorus, parathyroid hormone (PTH), and calcium levels. The patient’s phosphorous levels have remained normal with oral phosphorus supplementation.

## Discussion

TIO is a paraneoplastic syndrome that results in bone weakening from FGF-23 overproduction [7]. Phosphate is crucial in the formation of hydroxyapatite (calcium phosphate crystals), which is the inorganic portion of the bone matrix that gives bone its rigidity and strength [7]. Phosphate homeostasis is regulated by intestinal phosphate absorption, renal phosphate excretion, and the dynamic equilibrium between circulatory phosphate and phosphate in the calcified bone [7]. When serum phosphate levels fall too low, intestinal phosphate absorption is enhanced by calcitriol, the active form of vitamin D. When serum phosphate levels are higher than expected, renal phosphate excretion is enhanced through the increased expression of the sodium phosphate co-transporter located on the proximal tubules. Other hormones such as calcitonin and PTH regulate the dynamic equilibrium between circulatory phosphate and phosphate in the calcified bone.

FGF-23, yet another hormone, also plays a critical role in bone mineralization through the regulation of phosphate levels [7]. FGF-23 has a suppressive effect on renal 1α-hydroxylase resulting in impaired vitamin D hormone production leading to reduced intestinal absorption of phosphorus. FGF-23 also decreases the expression of the type 2a and type 2c sodium-phosphate co-transporters in the tubules resulting in decreased phosphate reabsorption [3]. As a result, the overproduction of FGF-23 results in hyperphosphaturia, hypophosphatemia, and low calcitriol levels.

FGF-23 overproduction is commonly associated with mesenchymal or mixed connective tissue tumors, hence the term tumor-induced osteomalacia [7]. Although rare, TIO should be suspected in patients presenting with consistent symptoms of bone pain, fractures, and muscle weakness as well as hypophosphatemia. If the latter is present, the patient should be evaluated for renal phosphate wasting. Once this is confirmed, additional lab workup can be done; this includes 1,25-dihydroxyvitamin D, calcium, and PTH levels. The measurement of FGF-23 is now also available and aids in the diagnosis of TIO [7,9]. While circulating FGF-23 levels are elevated in other hypophosphatemic conditions like autosomal dominant hypophosphatemic rickets, X-linked hypophosphatemic rickets, and autosomal recessive hypophosphatemic rickets, TIO can be distinguished from them by the presence of normal serum PTH and calcium levels with low or inappropriately normal 1,25-dihydroxyvitamin D levels [3,7]. However, PTH can be elevated in some cases due to secondary hyperparathyroidism in response to an elevated FGF-23 level [7].

The treatment of TIO involves the localization and surgical removal of the tumor [11,12], which promotes the reversal of the clinical and biochemical effects of FGF-23 overproduction [7]. The detection of these tumors, however, is often difficult due to their small size and slow growth, which may not be picked up on physical exam or imaging. This, as well as the rarity of the condition, often results in delayed diagnosis and treatment [9,11]. The average time from the onset of symptoms to a correct diagnosis often exceeds 2.5 years [7]. Once the diagnosis is made, the inability to localize the underlying tumor further delays definitive treatment by an average of five years [7]. The localization of tumors missed by computer tomography (CT), MRI, or even positron emission tomography (PET)-CT may be enhanced by the use of somatostatin-aided scintigraphy [7]. Surgical removal of the tumor is the only definitive treatment [9,11]. However, while patients have a good prognosis following surgical resection, recurrence may occur, affecting approximately 7% of patients who undergo surgery [7,9]. Metastasis at distant sites is also a possible sequela [11,12]. Patients require continuous follow-ups after surgery to monitor tumor recurrence or metastasis [11].

For individuals whose tumor cannot be resected or detected, management involves phosphate repletion and calcitriol supplementation. This typically includes elementary phosphorus at 30-60 mg/kg/day, divided in four to six doses, and calcitriol dosing approximately 2 mcg daily [9]. Because this treatment regimen increases the risk of secondary hyperparathyroidism and nephrolithiasis, burosumab is now considered the preferred form of therapy [7,13]. This is a recently approved monoclonal antibody that binds to FGF-23 blocking its effects downstream. It is well tolerated and has been shown to normalize serum phosphate concentrations, improve ambulation, and reduce pain [13].

As mentioned previously, due to the risk of recurrence or metastasis after surgical management, and secondary hyperparathyroidism or nephrolithiasis with medical management, frequent follow-ups with biochemical testing every three to six months are cardinal in these patients. Serum phosphorus, calcium, alkaline phosphatase, PTH, and 24-hour urine calcium are routinely measured. Phosphate and calcitriol supplementation should be tailored to maintain phosphorus within a low-normal range, without causing hypercalcemia or hypercalciuria [7,8,10]. FGF-23 levels are not required for long-term follow-ups unless the patient develops symptoms or persistent hypophosphatemia suggesting a recurrence of the tumor. However, FGF-23 levels are measured in the first follow-up visit after surgery to ensure adequate resection of the tumor. FGF-23 and serum phosphorus typically return to normal levels within the first five days post-resection [7].

Another important consideration of an FGF-23 inhibitor is its potential cardiovascular (CV) benefit. Increased FGF-23 levels may be a risk factor for increased CV morbidity and mortality [14-17]. This is thought to be due to the activation of myocardial FGF-23 receptors leading to left ventricular hypertrophy and cardiomyopathy [14,15,17,18], increasing the risk of sudden cardiac death [16]. It is a strong predictor for heart failure decompensation [14]. FGF-23 has been associated with endothelial dysfunction and inflammation and is shown to predict acute atherosclerotic CV events [14,15,16]. Decreased vitamin D levels and high phosphorous levels are independently associated with CV disease risk and mortality [16,19,20]. It may be that higher FGF-23 levels may also lead to a higher CV disease risk by additional mechanisms of vitamin D deficiency and altered phosphorus metabolism [19]. A meta-analysis by Marthi et al. demonstrated that a high FGF-23 concentration was associated with a 30% increased risk of myocardial infarction and stroke, 40% increased risk of CV mortality, and 50% increased risk of heart failure [18]. The Heart and Soul study demonstrated the possible FGF-23 effect on CV disease events and mortality among persons with a range of kidney function from normal to moderate chronic kidney disease (CKD), concluding that higher FGF-23 is independently associated with mortality and CV events [17]. While the FGF-23 effect on CV outcomes in patients with CKD has been studied in the literature, this has not been discussed in patients with TIO thus far.

## Conclusions

TIO is a rare paraneoplastic syndrome, which can cause significant morbidity if not diagnosed and treated early. Due to its nonspecific symptoms at presentation, difficulty in tumor localization, and rarity of the condition, diagnosis is often delayed. A high index of suspicion is required for the prompt diagnosis and management of this potentially curable condition. There may be a possible effect on cardiovascular morbidity and mortality associated with increased FGF-23 levels; however, this has not been currently studied in patients with TIO.
